# Long-COVID: neurological manifestations and management

**DOI:** 10.1007/s00415-021-10847-5

**Published:** 2021-10-21

**Authors:** Sioned Williams, Ray Wynford-Thomas, Neil P. Robertson

**Affiliations:** 1 Department of Neurology, University Hospital of Wales, Heath Park, Cardiff, CF14 4XN UK; 2 Department of Neurology, Division of Psychological Medicine and Clinical Neuroscience, Cardiff University, University Hospital of Wales, Heath Park, Cardiff, CF14 4XN UK

## Introduction

At the time of writing there have been over 110 million reported cases of COVID-19 across the world, since the first reports emerged in December 2019. Although there have also been nearly 3 million related deaths, the majority of patients recover without sequelae. However, a proportion who recover from the acute infection develop more persistent symptoms which vary in severity, duration, and phenotype; a phenomenon now known as Long-COVID. The underlying pathogenesis of Long-COVID remains unclear, but clinical symptoms can be extensive, affect multiple organs, and may persist for many months after illness-onset. Neurological symptoms may be prominent in Long-COVID and neurologists, in common with other medical specialists, are likely to require an understanding of relevant symptoms to develop optimal management strategies and services. In the UK, the National Institute for Health and Care Excellence defines Long-COVID as ‘signs and symptoms that continue or develop after acute COVID-19’. This includes both ongoing symptomatic COVID-19 (from 4 to 12 weeks post initial infection) and post-COVID-19 syndrome (12 weeks or more). In an effort to keep pace with published work, we review recently published studies on Long-COVID, with a focus on neurological features.

## Long-term coronavirus disease 2019 complications in inpatients and outpatients: a 1-year follow-up cohort study

Several studies have now depicted the short- and medium-term sequelae of COVID-19 in hospitalised or non-hospitalised cohorts. This study considers the clinical consequences of those with confirmed SARS-COV-2 infection, 12-months after recovery from acute illness. 303 participants (54% female) were consecutively recruited to a semi-structured phone interview to evaluate the presence of 37 persistent symptoms, according to those most frequently reported in recent literature. Symptoms were categorised as: respiratory disorders, fatigue and weakness, muscle and joint pain, movement impairments, neurological and cognitive impairments, sensory alterations, sleep disorders, and gastrointestinal symptoms. Patient reported symptoms were only recorded if they had not been present prior to acute infection with SARS-CoV-2. Regardless of the severity of the acute phase, most patients (81%) reported at least one symptom persisting 12 months after recovery from acute illness. Most prevalent symptoms were fatigue and weakness (52%), muscle and joint pain (48%), sleep disorders (47%), and neurological and cognitive impairment (36%). A higher prevalence of persisting symptoms was noted in older age groups (47–58 years), as well as in female participants.

***Comment:*** This study suggests that the commonest symptomatic long-term neurological sequelae of COVID-19 are fatigue, myalgia, sleep disorders and cognitive impairment, with other symptoms including hyposmia and dysgeusia. To guide service planning and implementation it will be of value to establish profiles of those patients at greatest risk and how long symptoms persist. Although informative, this study has some limitations including lack of a control group, which prevents establishing causal association, and the potential for recall bias. Continuing high rates of COVID-19 suggest that future studies may benefit from using prospective monitoring to address these issues.

*Lombardo M et al. (2021) Open Forum Infect Dis. 8(8):ofab384.*

## Persistent symptoms in adult patients 1 year after coronavirus disease 2019 (COVID-19): a prospective cohort study

In this study, 96 participants who had confirmed COVID-19 and were managed as either an in- or out-patient at a single centre, were followed up for 12-months. The course of symptoms, laboratory and immunological parameters, and quality of life of participants at 5 and 12 months after acute infection, were analysed. Between 5 and 12 months, there was an increase in frequency of fatigue (41.7% to 53.1%, *p* = 0.043) and dyspnoea (27.1 to 37.5%, *p* = 0.041). No significant abnormality in inflammatory markers were identified in those with persistent symptoms. Similarly, SARS-CoV-2 antibodies demonstrated a significant decline over time which did not correlate to the presence of symptoms reported at follow-up at 5 or 12 months. However, at 12 months, the group of participants (43.6%) with a high ANA titre (≥ 1:160 versus < 1:160) had significantly higher symptom frequency. Females with ANA titres ≥ 1:160 also had a significantly higher frequency of concentration difficulties than those with lower titres (66.7% vs 26.1%, respectively; *p* = 0.003). However, this trend was not reflected in the male participant group. Raised ANA titres did not correspond to initial disease severity.

***Comment:*** When assessing patients in whom Long-COVID is suspected it would seem appropriate that laboratory investigations should first be directed at excluding other common diagnoses, including anaemia or thyroid dysfunction. To date, studies have failed to identify any significant laboratory abnormalities in those with post-COVID syndromes. However, this study found a higher prevalence of neurocognitive symptoms in those with an elevated ANA titre (≥ 1:160). Although it has been hypothesised that there may be an autoimmune component of Long-COVID, the significance of raised ANA titres is currently unclear and further research is required to fully elucidate this. Limitations of this study include the relatively small cohort size and potential selection bias, reducing the generalisability of the results.

*Seeßle J et al. (2021) Clin Infect Dis. ciab611.*

## Post-COVID-19 syndrome (long haul syndrome): description of a multi-disciplinary clinic at mayo clinic and characteristics of the initial patient cohort

This study describes an established multi-disciplinary COVID-19 Activity Rehabilitation Programme (CARP) at Mayo Clinic, which serves to evaluate and manage patients with post-COVID syndrome. The paper depicts their first cohort of 100 patients (mean age, 45.4 ± 14.2 years; 68% women), with a mean of 93 days (range 28–159) following acute infection. Within the cohort, the most common symptoms were fatigue (80%), respiratory problems (59%), neurological complaints (59%), as well as subjective cognitive impairment and sleep disturbance. CARP interventions were delivered from 4 weeks after symptom onset or positive PCR, beginning with an initial evaluation to assess the individual’s symptoms, as well as the goals for their care. Interventions were directed at fulfilling the clinic’s 3 primary aims. Firstly, they aimed to detect any evidence of deterioration during the early recovery phase, as previous research has shown a higher mortality within the first 60 days of discharge for those hospitalised with COVID-19 (e.g., due to thromboembolic events). The second objective was to improve function, as 34% of patients reported ongoing difficulties in performing activities of daily living. By utilising physiotherapy and occupational therapy, an individually paced programme for general rehabilitation and conditioning could be developed in response to a patient’s initial assessment. Other therapies included brain rehabilitation. Finally, CARP aimed to facilitate a safe return to work, as only one-third of patients had been able to return to full time employment. Routine laboratory work did not reveal significant abnormalities or aid diagnosis for the majority of patients. Autonomic reflex screens were performed in many patients in response to labile vital signs and symptoms, such as persistent tachycardia. 15 patients had completed autonomic reflex testing, and 12 tests indicated abnormalities. These patients were referred to neurologists and, where appropriate, managed with treatments such as propranolol, increased hydration and salt intake, and compression garments.

***Comment:*** It has been hypothesised that autonomic dysfunction, including orthostatic intolerance and sudomotor dysfunction, could contribute to various Long-COVID symptoms, such as fatigue and other neurocognitive features. Furthermore, up to 25% of patients with postural orthostatic hypotension syndrome can be found to have raised ANA titres. Whilst a vital part of managing those with Long-COVID includes a multi-disciplinary team approach including physiotherapy and occupational therapy, it may also be relevant to assess for autonomic dysfunction in addition and manage accordingly. There is a significant reduction in quality of life for those affected by Long-COVID, emphasising the importance of planning for effective rehabilitation services. Guidelines for service referral will be imperative to prevent overwhelming services and this is likely to become even more important as the effect of the emerging variants on Long-COVID is yet to be determined.

*Vanichkachorn et al. (2021) Mayo Clin Proc. 96(7):1782–1791.*

